# Enzyme - Switch sensors for therapeutic drug monitoring of immunotherapies

**DOI:** 10.1016/j.bios.2023.115488

**Published:** 2023-10-01

**Authors:** Emma Campbell, Hope Adamson, Declan Kohl, Christian Tiede, Christoph Wälti, Darren C. Tomlinson, Lars J.C. Jeuken

**Affiliations:** a School of Biomedical Science, University of Leeds, Leeds, LS2 9JT, United Kingdom; b Astbury Centre for Structural Molecular Biology, University of Leeds, LS2 9JT, United Kingdom; c School of Molecular and Cellular Biology, University of Leeds, Leeds, LS2 9JT, United Kingdom; d School of Electronic and Electrical Engineering, University of Leeds, LS2 9JT, United Kingdom; e Leiden Institute of Chemistry, Leiden University, PO Box 9502, 2300 RA, Leiden, the Netherlands

**Keywords:** Affimer protein, Therapeutic drug monitoring, Herceptin, Humira, Rituxan, Yervoy

## Abstract

Therapeutic monoclonal antibodies (TmAb) have emerged as effective treatments for a number of cancers and autoimmune diseases. However, large interpatient disparities in the pharmacokinetics of TmAb treatment requires close therapeutic drug monitoring (TDM) to optimise dosage for individual patients. Here we demonstrate an approach for achieving rapid, sensitive quantification of two monoclonal antibody therapies using a previously described enzyme switch sensor platform. The enzyme switch sensor consists of a β-lactamase – β-lactamase inhibitor protein (BLA-BLIP) complex with two anti-idiotype binding proteins (Affimer proteins) as recognition elements. The BLA-BLIP sensor was engineered to detect two TmAbs (trastuzumab and ipilimumab) by developing constructs incorporating novel synthetic binding reagents to each of these mAbs. Trastuzumab and ipilimumab were successfully monitored with sub nM sensitivity in up to 1% serum, thus covering the relevant therapeutic range. Despite the modular design, the BLA-BLIP sensor was unsuccessful in detecting two further TmAbs (rituximab and adalimumab), an explanation for which was explored. In conclusion, the BLA-BLIP sensors provide a rapid biosensor for TDM of trastuzumab and ipilimumab with the potential to improve therapy. The sensitivity of this platform alongside its rapid action would be suitable for bedside monitoring in a point-of-care (PoC) setting.

## 1 Introduction

Detection of antibodies has high diagnostic value and can indicate many disease states including autoimmune disorders, allergies and infectious disease (Chevaliez et al., 2016; Hamilton and Franklin Adkinson, 2004; Zhang et al., 2014). Furthermore, the growing market of immunotherapies has provided a new purpose to antibody detection: therapeutic drug monitoring (TDM). This type of monitoring is usually seen alongside any drug treatments that have severe adverse reactions (SARs) and a narrow therapeutic window (Luque-Uría et al., 2021). Anticoagulant, immunosuppressive, and cytotoxic drugs are among those that need vigilant monitoring (Jossen and Dubinsky, 2016; Konkle, 2016; Paci et al., 2014). Monoclonal antibody (mAb) therapies are generally used as immunosuppressive or immune targeting agents in the treatment of autoimmune diseases and certain cancers (Du et al., 2017; Hansel et al., 2010; Scott et al., 2012). Although therapeutic mAbs (TmAbs) are generally well tolerated, SARs have been observed (Table 1) – attributed to their promiscuous pharmacological profile and the abundance of target receptors throughout the body (Hunt et al., 1992; Press et al., 1990). Interpatient variability of TmAb pharmacokinetics plus an association between inadequate serum mAb concentration and lack of therapeutic response means that TDM is necessary (Oude Munnink et al., 2016). Monitoring of trough concentrations of ipilimumab between dosages has improved survival of metastatic melanoma by preventing drug concentrations from dropping too low (Feng et al., 2013).

**Table 1:** Profiles of four mAb therapies and their associated side effects.

TmAb name (Brand Name)	Target	FDA approval	Indications for use	Adverse effects	Reference
Trastuzumab (Herceptin)	Human Epidermal growth factor receptor 2 (HER2)	1998	• HER2-positive breast carcinoma	• Cardiotoxicity with anthracycline • Pulmonary toxicity • Hypomagnesaemia	(Goldenberg, 1999)
Adalimumab (Humira)	Tumour necrosis factor-α (TNF- α)	2002	• Rheumatoid arthritis • Ankylosing spondylitis • Crohn’s disease • Ulcerative colitis	• Anaemia, leukopaenia & thrombocytopaenia • Malignancy, lymphoma & lymphoproliferative disorders	(Zhao et al., 2018)
Rituximab (Rituxan)	CD20 on B cells	1997	• Follicular non-Hodgkin’s lymphoma • CD20 diffuse large B cell non-Hodgkin’s lymphoma	• Cytokine release syndrome • Tumour lysis syndrome • Serum sickness • Progressive multifocal leukoencephalopathy	(Smith, 2003)
Ipilimumab (Yervoy)	Cytotoxic T lymphocyte antigen 4	2011	• Advanced renal cell carcinoma • Metastatic melanoma • Metastatic colorectal cancer	• Enterocolitis • Erythematous • Pruritus • Inflammatory hepatitis • Hypophysitis • Neuropathies	(Hansel et al., 2010)

Trastuzumab – a recombinant IgG1 kappa, humanized monoclonal antibody that binds to the extracellular domain of HER-2. Used to prevent excessive activation of proliferation pathways (Ras/Raf/mitogen-activated protein kinase (MAPK)) which lead to tumour growth. adalimumab inhibits TNF-α interacting with p55 and p75 cell surface TNF receptors, preventing downstream inflammatory pathways overly activated in autoimmune disorders. rituximab mediates B-cell lysis via complement dependent cytotoxicity (CDC) and/or antibody dependent cell-mediated cytotoxicity (ADCC). The exact mechanism is still unclear (Smith, 2003). ipilimumab inhibits CTLA4 which activates anti-tumour immunity and sustains T-cell activity (Hansel et al., 2010).

The most commonly used methods of measuring drug concentration currently in clinical use are the fluorescence polarization immunoassay (FPIA), liquid chromatography–tandem mass spectrometry (LC-MS/MS), enzyme immunoassay (EMIT), and enzyme linked immunosorbent assay (ELISA) (El Amrani et al., 2016; Schmitz et al., 2016; Steijns et al., 2002). Although highly sensitive and specific techniques, the lengthy timeframe to results makes them inadequate as a point-of-care (PoC) test. To address these limitations, rapid detection methods for the measurement of serum TmAb concentrations are being researched and developed (Bian et al., 2018; Ordutowski et al., 2022). A PoC platform would facilitate constant monitoring of therapeutic mAb titres so dose adjustments can be made to improve efficacy and quality of life (Chatelut et al., 2021).

**Fig. 1:**
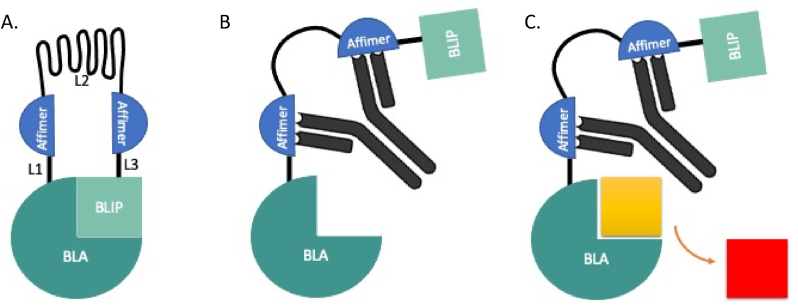
**The mechanism of detection by which BLA-BLIP senses monoclonal antibodies in a 1-pot, wash free assay.** (A) The BLA-BLIP sensor in its closed state with β-lactamase inhibitor protein (BLIP) associated with the enzyme (BLA). Affimer proteins are attached to the enzyme and inhibitor via rigid (L1 & L3) and semi-flexible (L2) peptide linkers. (B) Introduction of the analyte to the assay allows specific binding of the recognition elements to the mAb paratopes causing a conformational change in the enzyme switch sensor. BLA and BLIP dissociate from one another and the active site of BLA is free. (C) Nitrocefin is added to the assay solution and hydrolysed by the active BLA causing a colorimetric reaction from yellow to red. The change in absorbance measurement directly corresponds to the amount of free BLA and ergo the concentration of analyte.

Recent advances in molecular recognition techniques have drawn focus to engineered chimeric proteins for the detection of antibodies in biological fluids (Arts et al., 2016; Mocenigo et al., 2020; van Rosmalen et al., 2018). Allosteric enzyme switches provide a rapid platform for enzyme linked detection of clinically relevant proteins (Adamson et al., 2019a; Adamson and Jeuken, 2020; Guo et al., 2019; Nicholes et al., 2016; Vallée-Bélisle and Plaxco, 2010). A protein switch incorporating β-Lactamase (BLA) and its inhibitor protein (BLIP) has previously been shown to produce a readable signal within 15 min. The signal is produced in response to target driven disruption of a linked enzyme-inhibitor complex, where binding of both recognition elements results in a conformational change within the BLA-BLIP sensor (Fig. 1). Initial BLA-BLIP designs used anti-Haemagglutinin mAb peptide epitopes (Banala et al., 2013). Knowledge of epitope binding is necessary, when using them as recognition elements, which can rely on time consuming mapping techniques. The use of epitopes therefore limits the scope of targets that BLA-BLIP sensors can be developed against. Utilising the modular design of the BLA-BLIP enzyme-switch sensor, we recently showed that biomarker proteins can be detected down to pM concentrations when using Affimer proteins as recognition elements (Adamson et al., 2019a). Affimer proteins (∼12 kDa) are a class of non-immunoglobulin binders built on a cystatin scaffold with two highly variable regions that mediate molecular recognition (Tiede et al., 2017). These are particularly well suited to use in this type of system due to their small size and stability, making them easy to express within a multidomain construct.

Using the BLA-BLIP enzyme switch sensor with anti-idiotypic (anti-ID) Affimer proteins, we previously detected trastuzumab (Herceptin), a TmAb used in the treatment of HER2+ breast carcinoma (Goldenberg, 1999). Here, we expand on this work by determining technical challenges when this platform is adapted to other TmAb, providing a critical understanding of the requirements when this approach is more broadly applied (Adamson et al., 2019b). Including trastuzumab, all four TmAbs targeted (trastuzumab, ipilimumab, rituximab & adalimumab) are currently in clinical use for a range of treatments (Table 1).

## 2 Materials and methods

### 2.1 Materials

All restriction enzymes, buffers and cloning reagents were purchased from New England Biolabs LTD (NEB, Hitchin, UK), unless otherwise specified. All chemicals and reagents used were of analytical grade and purchased from Melford Biolaboratories Ltd. (Ipswich, UK) or Sigma Aldrich unless otherwise specified.

### 2.2 Generation of TmAb specific BLA-BLIP sensor constructs

All primers (Integrated DNA Technologies) used for cloning can be found in the supplementary material (Tables S3–6). We previously reported on anti-idiotype Affimer proteins raised against trastuzumab, ipilimumab, adalimumab and rituximab (Adamson et al., 2019b). Restriction cloning was used to introduce these anti-ID binding reagents into the BLA-BLIP sensor construct vector (based on the expression vector pET28a), as described previously (Adamson et al., 2019a). All DNA was purified using the Illustra GFX PCR DNA and Gel Band Purification Kit (GE Healthcare). Subcloned vectors were transformed into competent *Escherichia coli* XL-1 cells (Agilent Technologies). Charge Switch Pro Plasmid Miniprep Kit (Invitrogen) was used for all plasmid DNA purification and successful sub-cloning was confirmed by gene sequencing (sequences available in SI) of the full sensor constructs (GeneWiz).

### 2.3 Expression and purification of TmAb specific BLA-BLIP sensor constructs

All sensor constructs were expressed and purified using the protocol detailed by Adamson et al., (2019a). Briefly, plasmids (Table S7) containing the BLA-BLIP sensor constructs were transformed into *E. coli* BL21 (DE3) competent cells. 500 mL LB media (with 50 μg mL^−1^ kanamycin) was inoculated with a 10 mL starter culture and grown at 37 °C, 220 rpm. At OD_600_ ∼0.6, cultures were induced with 0.3 mM isopropylβ-D-thiogalactoside (IPTG) and grown overnight at 15 °C, 150 rpm. Cells were harvested and the periplasmic protein extracted by osmotic shock. The BLA-BLIP sensor construct was batch purified with Super Ni-NTA resin (Generon) via the N-terminal 6xHis tag. Eluates were pooled and further purified via the C-terminal Strep-II tag using the Strep-Tactin spin column kit (IBA). The double tag purified protein was buffer exchanged into storage solution (50 mM Tris, 150 mM NaCl, pH 7.4) using Zeba spin desalting columns (Thermofischer). Protein concentration was determined by BCA assay and purity checked by SDS-PAGE (Fig. S1). Aliquots were stored at −80 °C.

### 2.4 Characterisation of sensor functionality

#### 2.4.1 Targets

mAb biosimilars (Invivogen) that the anti-ID Affimer proteins were raised against were used as antibody targets, as follows: Anti-HER2Tra-hIgG4 (trastuzumab), Anti-hTNF-a-hIgG1 (adalimumab), AntihCTLA4-hIgG1 (ipilimumab) and Anti-hCD20-hIgG4 (rituximab). For the purposes of this work, they will be referred to by name of the TmAb.

#### 2.4.2 ELISA

40 μl of 20 μg mL^−1^ mAb in PBS (137 mM NaCl, 2.7 mM KCl, 8 mM Na_2_HPO_4_, 2 mM KH_2_PO_4_, pH 7.4) was adsorbed onto a Nunc Maxisorb microtiter plate for 1 h at 20 °C. After washing thrice with PBST (137 mM NaCl, 2.7 mM KCl, 8 mM Na_2_HPO_4_, 2 mM KH_2_PO_4_, 0.1%Tween-20, pH 7.4), wells were blocked with casein blocking buffer (Sigma-Aldrich) diluted 1:10 in PBST for 1 h and washed thrice with PBST. 40 μl of 10 μg mL^−1^ sensor (diluted in casein block) was applied to the plate for 1 h and washed with PBST before adding 25 μl 1 μg mL^−1^ Streptactin-HRP (IBA) in casein block for 1 h. Wells were washed 6 times before adding 50 μl 3,3′,5,5′-Tetramethylbenzidine (TMB) (Sigma-Aldrich) to the plate and incubated for 15 min at 20 °C. Absorbance was measured at 650 nm on a plate reader (MultiSkan FC, Thermo).

#### 2.4.3 BLA-BLIP assay

For all enzyme activity assays, non-binding surface 96-well plates (CORNING) were used, with a final volume of 200 μl assay buffer (50 mM sodium phosphate, 100 mM NaCl and 1 mg mL^−1^ BSA, pH 7). 2 nM of sensor were incubated with serial dilutions of their TmAb target for 15 min. Nitrocefin (Merck) was added at 50 μM and absorbance measured at 551 nm on a plate reader (MultiSkan FC, Thermo) after 10 min. Sensor and substrate concentrations, and incubation times were identical for all BLA-BLIP assays performed. A four-parameter logistic (4 PL) regression was fit to dose response curves using GraphPad Prism 9 software. Limit of detection (LoD) was calculated using the equation:

$$LoD=mean\;blank+1.645\left({SD}_{blank}\right)+1.645\left({SD}_{low\;conc.test}\right)$$

Where SD_blank_ refers to the standard deviation calculated when no analyte is present and SD_lowconc.test_ refers to the standard deviation calculated at the lowest concentration of analyte tested, in this instance 1 pM (Armbruster and Pry, 2008).

When measuring the activity of an enzyme, correcting background interference is commonplace with the subtraction of background signal to produce fold-gain measurements (Shapiro et al., 2009). The fold-activity gain of the BLA-BLIP sensors with target compared to without was calculated using:

$$\frac{A_{551}\left(target,t=10\right)―A_{551}\left(target,t=0\right)}{A_{551}\left(zero\;target,t=10\right)―\;A_{551}\left(zero\;target,t=0\right)}=\frac{{\Delta A}_{551}\left(target\right)}{{\Delta A}_{551}\left(zero\;target\right)}$$

Where A_551_(target) refers to absorbance measured at each concentration of analyte, A_551_(zero target) refers to the absorbance when no analyte is present, and *t* refers to the time (minutes) of the measurement.

For experiments testing stability in serum, pooled human serum (Clinical Trials Laboratory Services Ltd) was diluted in assay buffer at: 0%, 1% or 10% (v/v), and spiked with serial concentrations of TmAb.

#### 2.4.4 Cloning of cysteine residues onto anti-ID Affimer proteins

Cysteine residues were cloned onto the C-terminus of all anti-ID binding reagents for maleimide biotinylation to facilitate Streptavidin HRP detection within the bridge ELISA format (Fig. S2). Anti-ID Affimer protein DNA was amplified with primers to introduce C-terminal cysteine residues (Table S6) and cloned into pET11a plasmids via restriction cloning as previously described (Shamsuddin et al., 2021). Successful sub-cloning was confirmed by gene sequencing and correct clones transformed into competent BL21Star™ (DE3) for expression.

#### 2.4.5 Production and purification of anti-ID Affimer proteins

Affimer proteins were produced as previously described (Adamson et al., 2019b). Cells from a 50 mL culture were harvested by centrifugation at 4000×*g* for 15 min re-suspended in 1 mL lysis buffer (50 mM NaH_2_PO_4_; 300 mM NaCl; 30 mM Imidazole; 10% Glycerol; pH 7.4) supplemented with 0.1 mg mL^−1^ Lysozyme (Sigma-Aldrich, USA); 1% Triton X-100; 10 U mL^−1^ Benzonase® Nuclease (Merck, Germany); 1x Halt protease inhibitor cocktail. The solution was incubated at 20 °C for 1 h on a Stuart SB2 fixed speed rotator. The lysate was heat-treated at 50 °C for 20 min in a water bath and centrifuged at 16000×*g* for 20 min to remove cell debris and insoluble, heat denatured proteins. For each Affimer protein, the supernatant from the lysed cells was incubated with Ni-NTA resin for 1 h on a rotator at 20 °C. The resin was washed with wash buffer (50 mM NaH_2_PO_4_; 500 mM NaCl; 20 mM Imidazole; pH 7.4). Affimer protein was eluted from the resin with elution buffer (50 mM NaH_2_PO_4_, 500 mM NaCl; 300 mM Imidazole; 20% Glycerol; pH 7.4). All eluted samples were run on a 15% SDS-PAGE gel to monitor the purity of the protein.

#### 2.4.6 Biotinylation of anti-ID Affimer proteins

Prior to biotinylation of the Affimer proteins, they were desalted and exchanged into PBS using Zeba Spin Desalting Columns, 7K MWCO (Thermo Fisher) according to the manufacturer’s instructions and then diluted down to 0.5 mg mL^−1^ in PBS. Potential disulphide bonds were reduced with TCEP disulphide reducing gel according to manufacturer’s instruction (ThermoFisher Scientific). Cys-Affimer proteins were then biotinylated as previously described (Shamsuddin et al., 2021). Biotinylation was confirmed by a direct ELISA using streptavidin HRP. Final concentration of the biotinylated Affimer proteins were determined using the adsorption at 280 nm (A_280_) using a DS-11 Nanodrop spectrophotometer and using a BCA protein assay (Smith et al., 1985).

#### 2.4.7 Bridge ELISA

A Nunc Maxisorb 96-well plate was first coated with capture Affimer protein (1 μg mL^−1^) and incubated for 1 h at 20 °C. After washing thrice with PBST (0.2% Tween-20), 50 μL casein blocking buffer (1:10 in PBST) was incubated in each well for 1 h at 20 °C, followed by washing thrice with PBST. Increasing concentrations from 10 ng mL^−1^ to 30 μg mL^−1^ of each of the TmAb were incubated in each well for 1 h at 20 °C followed by washing thrice with PBST. The biotinylated detection Affimer protein was then added to the plate (2 μg mL^−1^) and incubated for 1 h at 20 °C, followed by washing thrice with PBST. Streptavidin HRP (1 μg mL^−1^) was then added to the wells and incubated at 20 °C for 30 min. This step was followed by washing 6x with PBST and incubation with 50 μL of TMB for 15 min, the plate was then read at 650 nm using a plate reader.

#### 2.4.8 Data analysis

All data are presented as the mean of at least 3 biological repeats with error bars representing ± Standard Error of the Mean (SEM). To determine the significance of data, one-tailed, homoscedastic t-tests were used. Significance was shown by p<0.05.

The quantifiable ranges of the sensors were determined based on parameters set by the food and drug administration (FDA) for validation of new bioanalytical methods (Administration and (CDER) 2018), including percentage recovery values between 80 and 120% and the coefficient of variation (% CV) < 25%.

## 3 Results

### 3.1 Optimisation of signal change in anti-trastuzumab BLA-BLIP sensor

To create the anti-ID sensor constructs, two copies of anti-ID Affimer proteins, previously described (Adamson et al., 2019b), were attached to TEM1–β-lactamase (BLA) and β-lactamase inhibitor protein (BLIP) using recombinant DNA technology. Surface plasmon resonance (SPR) analysis showed low nM affinity values for all four anti-ID binding reagents (Table S1).

**Fig. 2:**
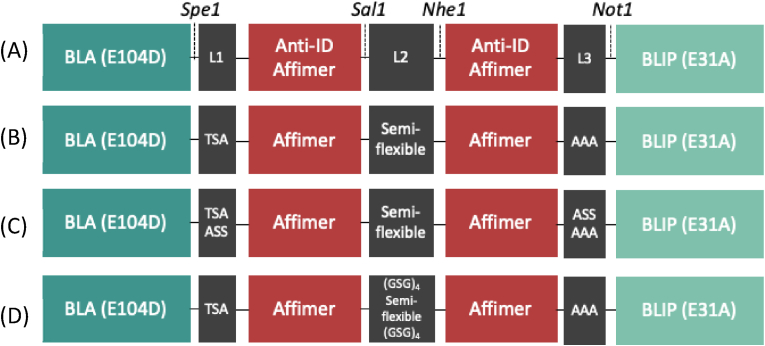
**Schematic diagrams of sensor components (A)** The general BLA-BLIP sensor construct (BB_anti-ID) **(B)** BB_Trast as previously reported (Adamson et al., 2019a) where the semi-flexible linker is (GSG)_6_A(EAAAK)_6_A(GSG)_6_A(EAAAK)_6_A(GSG)_6_**(C)** BB_Trast2 with Longer L1 and L3 linkers **(D)** BB_Trast3 with additional (GSG)_4_ encompassing the semi flexible linker to give a longer L2 linker of (GSG)_10_A(EAAAK)_6_A(GSG)_6_A(EAAAK)_6_A(GSG)_10_.

The mechanism of target driven disruption employed by BLA-BLIP to produce a signal is largely maintained by recognition element affinity and the three peptide linkers between the four protein domains (Fig. 1, Fig. 2a). It has previously been reported that the use of two specific recognition elements within this sensor design is essential for enzyme-inhibitor disruption, with only one specific recognition elements unable to produce a signal (Adamson et al., 2019a). Linker 1 (L1) and linker 3 (L3) anchor the two recognition elements to BLA or BLIP, respectively. These linkers need to be short enough to aid the disruption of the enzyme-inhibitor complex, but long enough to not impair folding of the protein domains. Linker 2 (L2), in contrast, needs to be sufficiently long to allow the recognition elements to bind both variable regions of the TmAb (Fig. 1). Point mutations were made at the interface of BLA (E104D) and BLIP (E31A) to weaken their interaction (Adamson et al., 2019a), facilitating dissociation of the complex in response to binding of both recognition elements. The addition of a chromogenic compound, hydrolysed by BLA, allows the activity of the enzyme to be determined by measuring light adsorption at 551 nm, which directly corresponds to the concentration of analyte, in this case TmAbs.

We previously reported a proof-of-principle of the anti-trastuzumab BLA-BLIP sensor (Adamson et al., 2019a), but did not optimise the linkers (L1, L2 and L3, Fig. 2) for sensor performance. Linker optimisation has previously helped improve sensor activity when adapting to new targets, especially when Affimer binding sites on the target analyte are unknown. To optimise the overall signal change, three constructs against trastuzumab (BLA-BLIP_trastuzumab) were compared with varying linker lengths (BB_Trast, BB_Trast2 and BB_Trast3; Fig. 2). BB_Trast was previously reported and has short, rigid L1 and L3 linkers (three amino acid residues) along with a semiflexible L2 linker – (GSG)_6_A(EAAAK)_6_A(GSG)_6_A(EAAAK)_6_A(GSG)_6_ – known to span the distance between the variable regions of mAbs (Adamson et al., 2019a; Banala et al., 2013). BB_Trast2 introduced longer L1 and L3 linkers (six amino acid residues) with the same semiflexible L2 linker. In BB_Trast3, the L2 linker was varied noting that monoclonal antibodies have a uniform shape with a fixed distance between the variable domains. The semiflexible L2 linker was thus only slightly lengthened to (GSG)_10_A(EAAAK)_6_A(GSG)_6_A(EAAAK)_6_A(GSG)_10._

The activity of BB_Trast, BB_Trast2 and BB_Trast3 in response to trastuzumab were measured as BLA activity using a homogenous assay where the turnover of nitrocefin can be measured as light absorbance at 551 nm (A_551_) (Fig. S3). BB_Trast outperformed BB_Trast3 in terms of fold-activity gain measured at A_551_ (p≤0.05). Additionally, the calculated LoD for BB_Trast3 was significantly increased at 3 nM compared to 300 pM for BB_Trast. BB_Trast2 has a calculated LOD of 300 pM, but a very low signal gain. All anti-ID BB_sensors from this point onwards were designed in the same format as BB_Trast (Fig. 2B).

### 3.2 Therapeutic monoclonal antibody detection

The recognition elements of BLA-BLIP sensors are easily exchanged genetically using the restriction sites flanking position A (*Spe* I & *Sal* I) and position B (*Nhe* I & *Not* I). This allowed us to create three anti-ID BLA-BLIP sensors with the anti-ID Affimer proteins against rituximab, adalimumab and ipilimumab (Adamson et al., 2019b) to produce the BLA-BLIP sensors BB_Rit, BB_Ada and BB_Ipi, respectively, alongside BB_Trast.

**Fig. 3:**
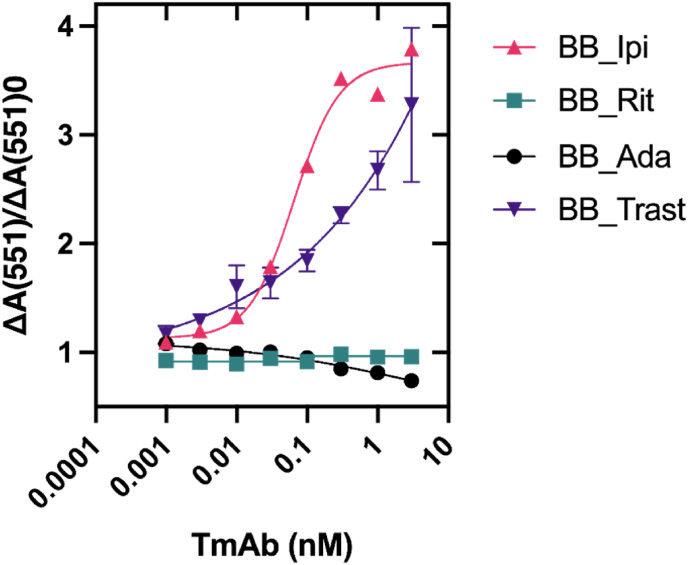
**The BLA-BLIP sensor construct can be applied to the detection of Ipilimumab when anti-idiotype Affimer proteins against the mAb are inserted.** Activity of BB_Trast, BB_Rit, BB_Ada and BB_Ipi when incubated with 0.001–3 nM of their respective TmAb analyte and presented as fold activity gain from the baseline (no analyte). All data are presented as a mean of at least three repeats and error bars represent ±SEM. Where error bars are not visible, they are situated within the symbol plot.

**Fig. 4:**
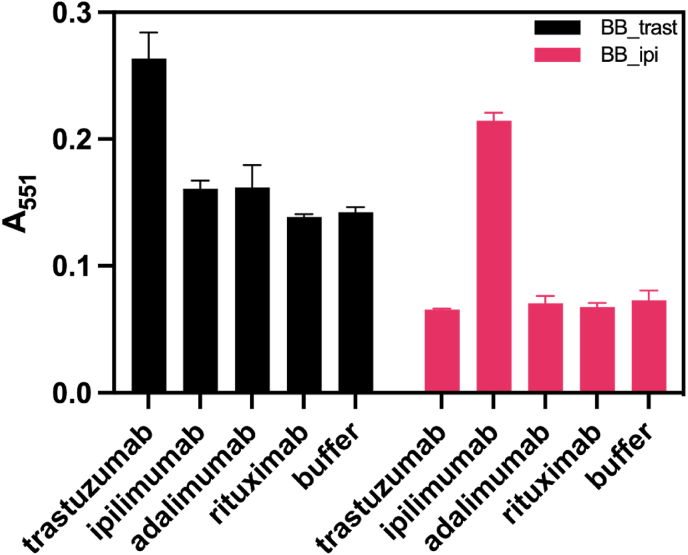
**TmAb BLA-BLIP sensors are specific to their target analyte**. 2 nM of the BLA-BLIP sensor constructs were incubated with 10 nM of specific TmAb, non-specific TmAb or blank buffer. BB_Trast and BB_Ipi both displayed significant (p ≤ 0.05) absorbance at A_551_ compared to non-specific TmAb analytes and blank buffer. Data of biosensors binding to their respective targets and non-specific analytes are presented as a mean of three repeats of raw A_551_ measurements and error bars represent ±SEM.

The dose response of BB_Trast, BB_Rit, BB_Ada and BB_Ipi were measured using the homogenous BLA-BLIP assay (Fig. 3). A pronounced “switch on” effect was displayed by BB_Trast and BB_Ipi when incubated with varying concentrations of TmAb. BB_Trast showed a 3.3-fold increase in activity in response to 3 nM trastuzumab, similar to the 2.8-fold increase previously reported (Adamson et al., 2019a). BB_Trast activity never reaches saturation across this concentration range (1 pM–3 nM). We avoided the use of TmAb concentrations >3 nM as a sensor concentration of 2 nM was tested and a “hook” effect would likely be seen at higher concentrations. This occurs when sensor components bind multiple analyte molecules, preventing disruption of the enzyme-inhibitor complex. BB_Ipi responded to 3 nM ipilimumab with a 3.5-fold increase in activity. The LoD of BB_Ipi was calculated at 30 pM, whereas the LoD of BB_Trast was calculated at 300 pM. BB_Ipi outperformed BB_Trast, both in terms of fold-activity gain at C_max_ and LoD. BB_Trast and BB_Ipi showed high specificity to their individual TmAbs. When incubated with 10 nM non-specific TmAbs in the BLA-BLIP assay (Fig. 4), there was no significant (p>0.05) response compared to the response generated in the presence of buffer only. Specificity of sensor binding was further demonstrated with direct ELISA (Figs. S4–S5).

### 3.3 Affimer protein – TmAb binding

No enzymatic response was observed from BB_Rit or BB_Ada in the presence of their respective TmAb target (Fig. 3). One explanation for this could be that the synthetic binding reagents against rituximab and adalimumab no longer bind to their targets once incorporated into the BLA-BLIP sensor constructs. To test whether the recognition elements were compromised in the BB_Rit and BB_Ada sensors, we performed direct ELISA on the BLA-BLIP sensor constructs (Fig. S4) using the C-terminal strep-II tag for detection.

**Fig. 5:**
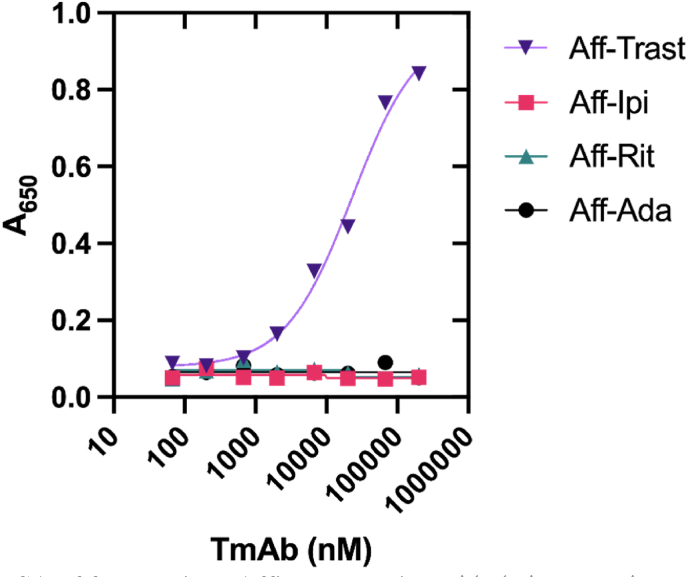
Bridge ELISA of four anti-ID Affimer proteins with their respective mAb targets presented as absorbance at 650 nm as a measurement of TMB turnover by HRP. All data are presented as a mean of three repeats with error bars representing ±SEM. Where error bars are not visible, they are within the symbol plot.

The direct ELISAs confirmed that BB_Rit and BB_Ada bound to their targets, even though the responses of the homogenous enzyme assay indicate that binding to their respective TmAbs does not result in the disruption of BLA-BLIP complex. This could be due to only one Affimer protein binding the TmAb instead of two as required to switch on the enzyme activity (see Fig. 1). To test whether two Affimer proteins can bind, we performed a bridge ELISA (Fig. 5; see Fig. S6 for a schematic of a bridge ELISA) (Adamson et al., 2019b; Townsend et al., 2016). Typically a pharmacokinetic (PK) assay, bridge ELISAs utilise anti-ID antibodies and take advantage of the two identical binding sites on IgG Abs to measure the concentrations of mAbs (Jani et al., 2016). As expected, the bridge ELISA confirms that two Affimer proteins can simultaneously bind to trastuzumab, whereas the binding reagents against rituximab or adalimumab were unable to detect their respective TmAbs in the bridge ELISA (Fig. 5). The latter could indicate that only one Affimer protein can bind to rituximab or adalimumab, preventing the switching mechanism to function as intended. The lack of response from the binding reagents against ipilimumab in this format is unexpected and is discussed below.

### 3.4 BLA-BLIP enzyme switch sensor performance in serum

**Fig. 6:**
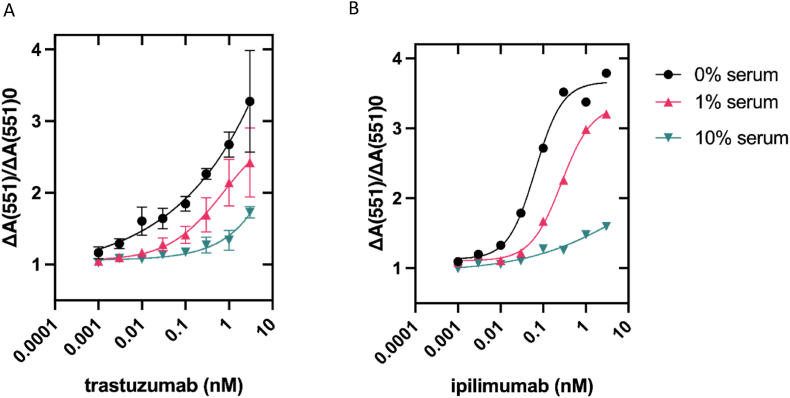
**BB_Trast and BB_Ipi maintain activity in 1% serum**. Serial dilutions of trastuzumab (A) and ipilimumab (B) were made up in 0%, 1% or 10% pooled human serum and incubated with the enzyme-switch sensors. Data plotted as fold activity gain from baseline measurements, as a mean of at least three repeats with error bars representing ±SEM. Where error bars are not visible, they are within the symbol plot.

The activity of BB_Trast and BB_Ipi were tested in the BLA-BLIP assay in varying percentages of pooled human serum (Fig. 6). The activity of BB_Trast in response to trastuzumab (Fig. 6A) was maintained when incubated in up to 1% serum, with no significant (p≤0.05) changes in fold-activity gain and a LoD of 300 pM retained. The stability in serum seen with BB_Trast was mirrored by BB_Ipi (Fig. 6B), although the LoD in 1% serum slightly worsened from 30 pM to 100 pM. Quantifiable ranges for BB_Trast and BB_Ipi were calculated in 1% human serum as 30 pM–3 nM and 30 pM–300 pM respectively (See Table S2 for details). BB_Trast and BB_Ipi were unresponsive to increasing concentrations of their respective analytes when in 10% serum. This is thought to be due to the breakdown of nitrocefin and inhibition of BLA by the variety of serum proteins including IgG and IgE antibodies present (Callaghan, 1978; Dalhoff and Brunner, 1983).

## 4 Discussion

One of the challenges when designing healthcare biosensors is performance in biological fluids. The complex composition of biological fluids, be that blood, saliva, or faeces, poses the problem of interference from nonspecific proteins. This is especially apparent with enzyme-based biosensors with many biological components able to interfere with enzymatic activity (Dimeski, 2008; Zhang et al., 2019). The implementation of TDM for mAb therapies has, so far, been affected by the difficulties presented when detecting mAbs in serum. Furthermore, with BB_Trast and BB_Ipi, the recognition elements are specific to humanized IgG1 kappa monoclonal antibodies (Goldenberg, 1999; Lipson and Drake, 2011), the antibody type most abundant in human sera (Hamilton, 2014). TmAb titres have been successfully monitored using PoC platforms where 1:100 dilutions of patient samples were necessary (Iria et al., 2022). Clinically relevant serum concentrations of trastuzumab are between 70 and 2000 nM and ipilimumab trough concentrations range between 30 and 230 nM (Koguchi et al., 2020; Leyland-Jones et al., 2010). BB_Trast and BB_Ipi can detect TmAb spiked buffer at concentrations 100x lower than the clinically relevant values. Furthermore, our results show that both sensors maintain their activity in 1% serum. This allows for dilution of patient serum to prevent matrix effects whilst maintaining the required sensitivity, thus both sensors presented here can measure clinically relevant concentrations of TmAbs within 30 min without the need for pre-treatment or washing steps such as those required in ELISAs. In HER2+ breast cancer, tumour shedding of the extracellular domain (ECD) of HER2 in exosomes can occur, especially in advanced disease (Ciravolo et al., 2012). These exosomes can bind circulating trastuzumab with an inhibitory effect, which should be accounted for in TDM methods (Damen et al., 2009). Free (unbound) drug concentrations are the most essential measurement for TDM as these are available for distribution and pharmacodynamic action (Wright et al., 1996). The recognition elements implemented in BB_Trast target the variable regions of trastuzumab and therefore we can speculate that exosome-bound trastuzumab would not be measured in this system, but HER2+ patient serum should be tested to evaluate discrepancies.

These enzyme-swich sensors have the capacity to be implemented into a doctor’s office or at a patient’s bedside for single measurements of trough concentrations before dose administration, to ensure maintenance of the minimal effective concentration (MEC) and allow dosing to be adjusted accordingly (Oude Munnink et al., 2016). For both trastuzumab and ipilimumab this would occur on a weekly, three-weekly, or monthly regimen (Feng et al., 2014; Hsieh et al., 2022; Levêque et al., 2008).

Current ELISA methods used for measurement of ipilimumab and trastuzumab have a lower limit of quantification (LLOQ) of 2 nM (Feng et al., 2014) and 1 nM (Cobleigh et al., 1999; Quartino et al., 2016) respectively. These ELISA methods require 1/100 dilutions of serum equivalent to our BLA-BLIP assay. Our BLA-BLIP assays can detect ipilimumab and trastuzumab at concentrations as low as 30 pM in 1% serum, which is an improvement on the sensitivity of current ELISA methods. Additionally, the much shorter, 30-min timeframe of our BLA-BLIP assay, compared to the 3–4 h timeframe of ELISA detection methods (Pegram et al., 1998) make the BLA-BLIP assay a prospective alternative to current TDM methods.

The BLA-BLIP sensor presented here is classified by its switching mechanism as a modular allosteric switch. Unlike proximity switches, also known as split enzyme systems, allosteric switches maintain the full activity and stability of their reporter both in an active and inactive state (Adamson et al., 2019a; Banala et al., 2013). Therefore, they do not suffer from the consequences of incorrect reassembly and subsequently compromised activity recovery (Guo et al., 2016; Su et al., 2018). This does, however, increase overall background activity, which must be accounted for. Other modular systems, similar to the one presented in this study, have also suggested a binding-induced conformational change as the driving force for sensor functionality (Golynskiy et al., 2010; Porchetta et al., 2018; Vallée-Bélisle and Plaxco, 2010). Allosteric switch biosensors require special attention to the linker lengths, specifically the distance between the recognition elements (Linker L2, Fig. 2). When using linkers to span IgG mAb variable regions, linker lengths significantly shorter or longer than the distance between these regions (10–12 nm) impacts the signal output. Using a consistent linker design between mAb targets has previously yielded versatile sensors (Porchetta et al., 2018), hence here we only made small changes to Linker L2. The original linker used in our previous proof-of-concept study (Fig. 2B) was a design based on a sensor construct with short epitope peptides as recognition elements (Banala et al., 2013). Here, it was reasoned that using the larger Affimer recognition elements might require a longer linker L2 (Fig. 2D), but instead the results show that the shorter, original linker gave a better performance.

Based on the data produced for BB_Rit and BB_Ada, showing a lack of signal in response to their respective TmAb analytes, we can speculate that there are issues with the binding of the recognition elements when incorporated into the BLA-BLIP enzyme switch, preventing them from functioning as intended. The schematic model of our enzyme – inhibitor switch sensor (Fig. 1) implies that the affinity of the recognition elements must be above the threshold necessary to successfully disrupt the BLA-BLIP interaction (K_i_ = 2.1 μM) (Banala et al., 2013). SPR analysis showed low nM affinity values for all four anti-ID binding reagents (Table S1). Although, these K_D_ values differ significantly (p<0.05) to at least one other anti-ID binding reagent analysed, they are all within 12-fold of one another and well above the threshold needed to disrupt the enzyme switch. The lack of correlation between affinity and sensor activity suggest that low affinity is not the primary cause of BB_Rit and BB_Ada inactivity.

The successful disruption of the enzyme inhibitor complex could be dependent on exact binding geometries of the recognition elements, which is a difficult factor to control and likely varies between targets. The binding sites on trastuzumab of Aff-Trast have since been determined with hydrogen–deuterium exchange mass spectrometry (HDX-MS) (Olaleye et al., 2023), and were as expected. Structural data on the binding of Aff-Rit and Aff-Ada to their respective targets could help explain the lack of activity from BB_Rit and BB_Ada. Previous work has confirmed that the BLA-BLIP switch mechanism requires two anti-ID binding proteins that interact with the variable regions of a TmAb to activate the reporter enzyme (Adamson et al., 2019a). The lack of response from BB_Rit and BB_Ada could thus be due to the inability of two Affimer proteins to bind simultaneously to the TmAb variable regions. Indeed, the negative bridge ELISA responses for rituximab and adalimumab are in line with this hypothesis. However, the bridge ELISA was also negative for the anti-ipilimumab binding proteins, while the BB_Ipi sensor is functional. We speculate that in the latter bridge ELISA, non-optimal orientation of the Affimer protein and ipilimumab on the polystyrene plate, which is known to affect sensitivity of immunodiagnostic procedures (Welch et al., 2017), prevents the assay from working adequately. Still, no conclusive correlations between bridge ELISA activity and successful BLA-BLIP sensors can be made and it is possible that BB_Ipi functions in an alternative, unknown mechanism. Either way, a screening method that directly tests whether two binding reagents can bind simultaneously would stimulate the development of BLA-BLIP sensor constructs, as current screening methods for high affinity binders do not always translate to binding proteins that are suitable for sensor development (Adamson and Jeuken, 2020; Peltomaa et al., 2019).

## 5 Conclusion

The simple “one pot” set up of the BB_Trast and BB_Ipi assays, which require no washing steps like those necessary in ELISAs, means the assay can easily be adapted into a diagnostic device for a point-of-care setting. This is in contrast to the standard tests currently used for measuring TmAb concentrations such as LC-MS/MS and ELISA which require skilled personnel and take multiple hours to produce results with LoD values in the low nM range (Berinstein et al., 1998; El Amrani et al., 2016; Quartino et al., 2016; Wolbink et al., 2005). Comparatively, the sub nM LoD and <45-min timeframe of our homogenous assay would allow for the immediate action needed for successful implementation of TDM.

The accurate monitoring of drug therapies with narrow therapeutic windows opens the door to personalisation of dosage, which can improve treatment success and quality of patient life (Taddeo et al., 2020). Due to the universal shape of TmAbs alongside the BLA-BLIP sensor mechanism of target driven disruption, we hypothesised easy adaptation of BB_Trast to three other TmAbs. A 50/50 split on successful implementation, suggested that the adaptation of BLA-BLIP to multiple TmAb targets is not as simple as expected, and optimisation of each sensor would be recommended. Overall, we presented two BLA-BLIP sensors that could detect clinically and therapeutically relevant TmAb concentrations with comparable sensitivity to current ELISA methods in 1% serum and significantly shorter run times, opening the opportunity for implementation in TDM.

## CRediT authorship contribution statement

**Emma Campbell:** Data curation, Formal analysis, Methodology, Writing - original draft. **Hope Adamson:** Methodology, Data curation, Writing - review & editing. **Christoph Wälti:** Conceptualization, Funding acquisition, Supervision. **Darren C. Tomlinson:** Methodology, Supervision, Methodology, Supervision. **Lars J.C. Jeuken:** Conceptualization, Funding acquisition, Methodology, Supervision, Writing - review & editing.

## Declaration of competing interest

The authors declare the following financial interests/personal relationships which may be considered as potential competing interests: The Affimer reagents used in this report are owned by the University of Leeds (UoL) but licensed to Avacta Life Sciences. The UoL receive royalties from Avacta Life Sciences as part of the license agreement, which is managed by the commercialisation team. The authors declare no competing financial interest.

## Data Availability

Data will be made available on request.
