# Supplementary material for: Enzyme - Switch sensors for therapeutic drug monitoring of immunotherapies
Source: Biosens Bioelectron. 2023 Oct 1;237:None. doi: 10.1016/j.bios.2023.115488 (PMC10427837; doi:10.1016/j.bios.2023.115488)
Supplement: Multimedia component 1 [file mmc1.docx]

**Supplementary Information**

Enzyme-Switch Sensor for Therapeutic Drug Monitoring of Immunotherapies

Emma Campbell^1,2^, Hope Adamson^1,2^, Modupe Ajayi^2,3^, Christoph Wälti^4^, Darren Tomlinson^2,3^, Lars JC Jeuken^1,2,5,*^

^1^ School of Biomedical Science, University of Leeds, Leeds, LS2 9JT, United Kingdom
^2^ Astbury Centre for Structural Molecular Biology, University of Leeds, LS2 9JT, United Kingdom
^3^ School of Molecular and Cellular Biology, University of Leeds, Leeds, LS2 9JT, United Kingdom
^4^ School of Electronic and Electrical Engineering, University of Leeds, LS2 9JT, United Kingdom
^5^ Leiden Institute of Chemistry, Leiden University, PO Box 9502, 2300 RA, Leiden, the Netherlands
* Corresponding author: L.J.C.Jeuken@lic.leidenuniv.nl

**Contents**

Supplementary Methods

Supplementary Figures

DNA and Protein Sequences

Table of Primers

Table of Plasmids

**Supplementary Methods**

Surface plasmon resonance (SPR)

Affimer affinities for their TmAb analyte were determined by surface plasmon resonance (SPR) using a BIAcore 3000 (GE Healthcare Europe GmbH). Trastuzumab, ipilimumab, adalimumab and rituximab were covalently immobilized on separate channels of a CM5 sensor chip with amine-coupling chemistry. The chip was activated with 200 mM 1- Ethyl-3-(3-dimethylaminopropyl) carbodiimide (EDC) and 50 mM N-Hydroxysuccinimide (NHS) before target injection under optimised conditions (trastuzumab, 5 µg ml-1 in 10 mM acetate pH 5.5; ipilimumab, 5 µg ml-1 in 10 mM acetate pH 5.5; rituximab, 5 µg ml-1 in 10 mM acetate pH 5.5; adalimumab, 5 µg ml-1 in 10 mM acetate pH 4.5). Remaining reactive groups were dcapped with ethanolamine (1 M, pH = 8.5). Biacore experiments were performed at 25°C in PBST buffer (PBS pH 7.4, containing 150 mM NaCl and 0.2 % Tween 20). Affimers were injected at 1.5625, 3.125, 6.25 and 12.5 nM at a flow rate of 5 μl min^-1^, followed by 12 minute dissociation. The on- and off- rates and Kd parameters were obtained from a global fit to the SPR curves using a 1:1 langmuir model, using the BIAevaluation software. Quoted Kd values are the mean ± SEM of three replicate runs, unless specified otherwise.

**Supplementary Figures**


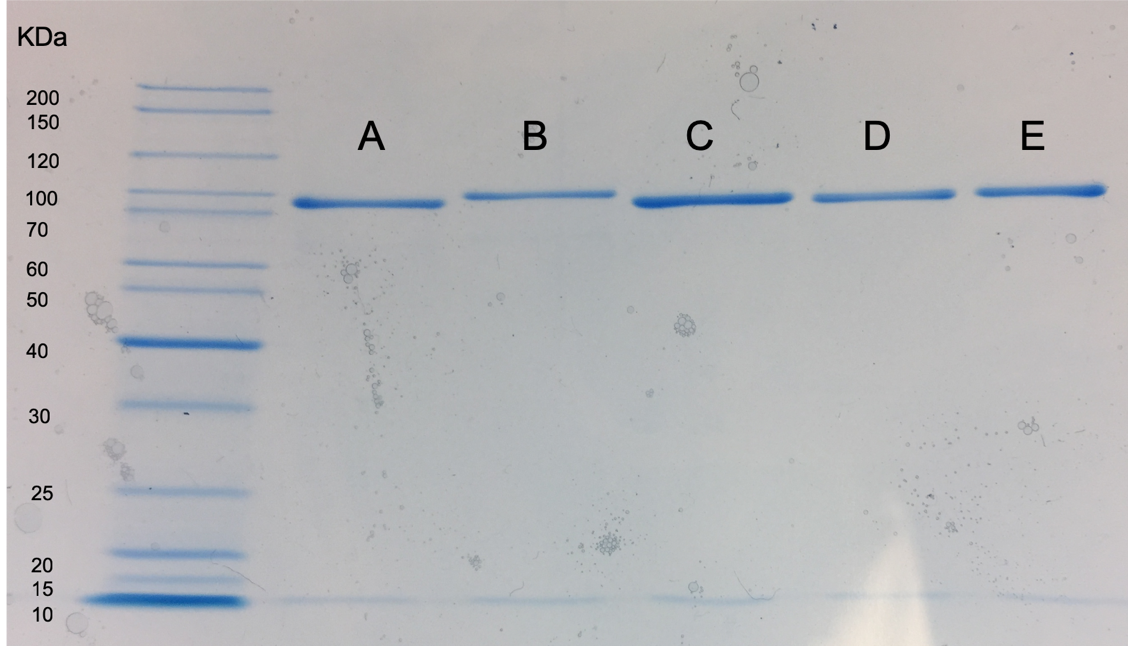


**Figure S1** SDS-PAGE of 5 BLA-BLIP constructs purified A: BB_Trast2, B: BB_Trast3, C: BB_Ipi, D: BB_Rit, E: BB_Ada. All constructs came out at ~80 kDa. Construct B however, had a slightly higher KDa (~83) due to the longer middle linker.


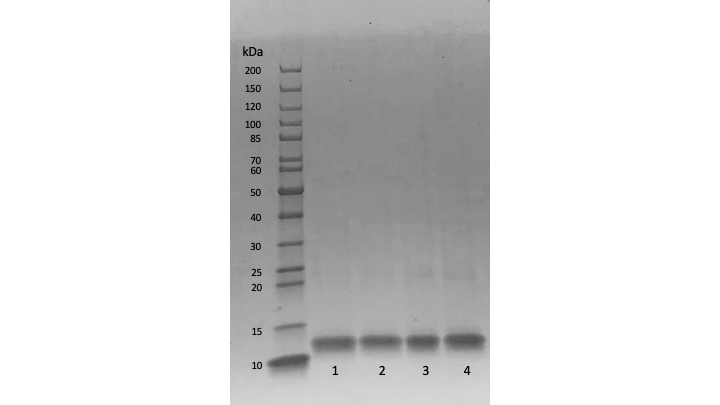


**Figure S2** 15% SDS PAGE of purified Cysteinated anti-ID Affimers: 1: Aff_Trast, 2: Aff_Ada, 3: Aff_Ipi and 4: Aff_Rit.


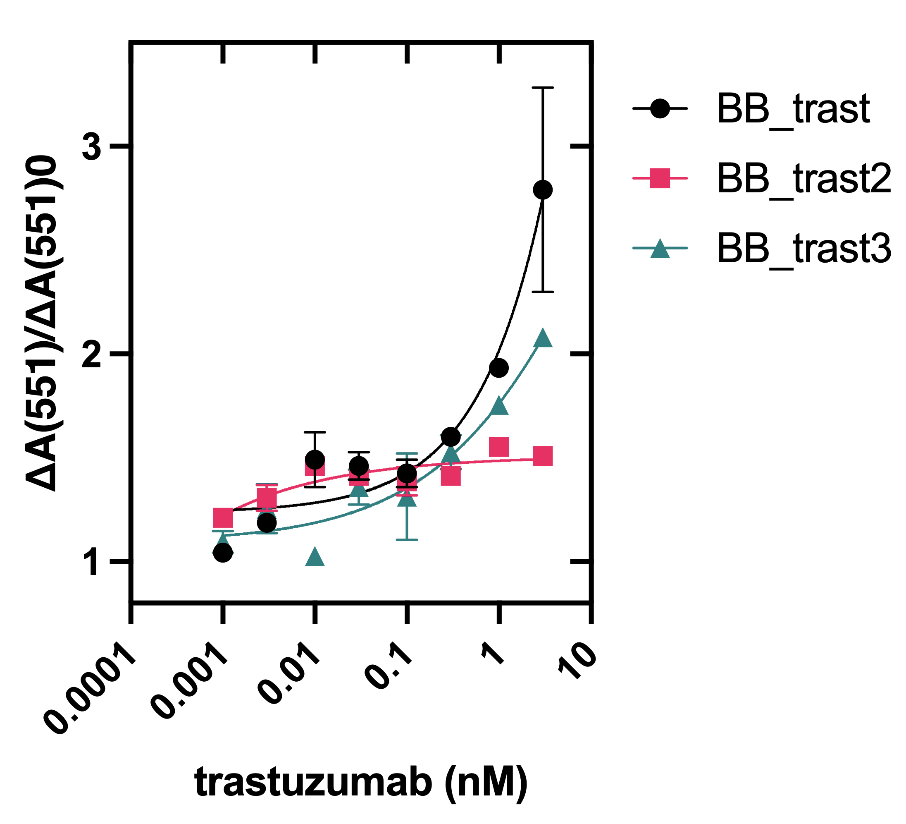


**Figure S3** **Length of linkers affects the BLA activity of the biosensor construct.** Activity of sensors: BB_Trast (TSA Trast Semi Trast AAA), BB_Trast2 (TSAASS Trast Semi Trast ASSAAA) and BB_Trast3 (TSA Trast (GSG)_4_ Semi (GSG)_4_ Trast AAA) were measured as absorbance at A_551_ and presented as fold activity gains ΔA_551_ (x nM Ab)/ΔA_551_ (0 nM Ab). All data are presented as a mean of at least three repeats with error bars representing ±SEM. Where error bars are not visible, they are situated within the symbol plot.


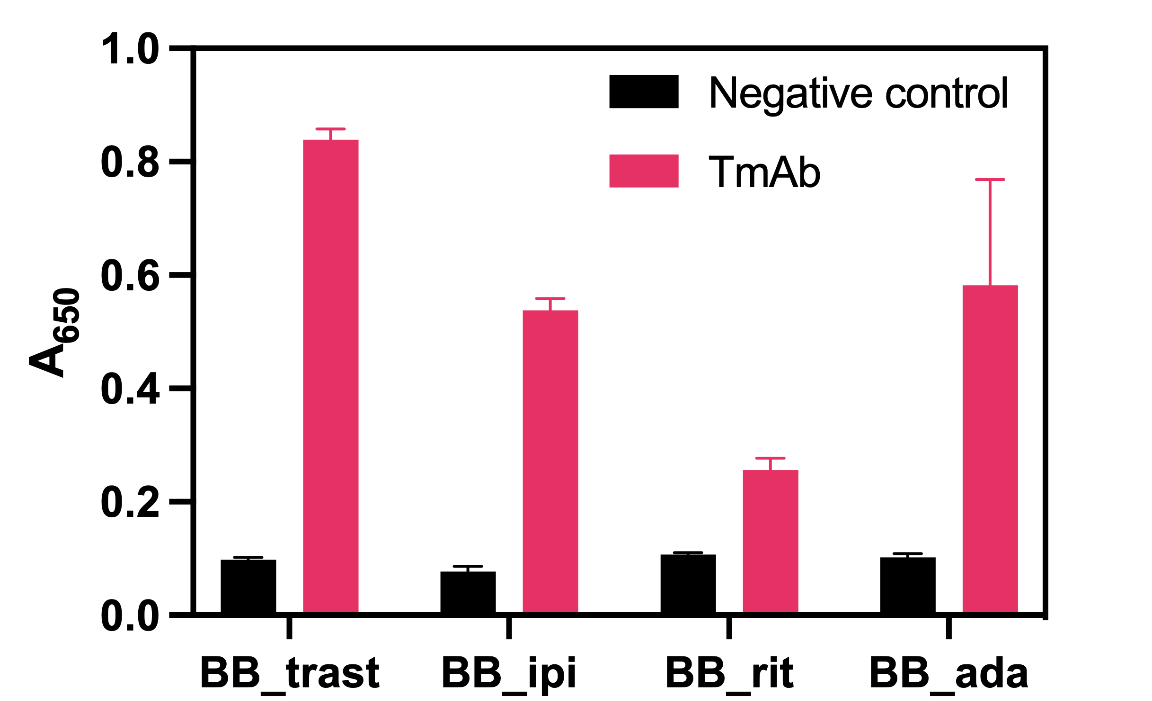


**Figure S4 Direct ELISAs of BB_sensors.** ELISA data demonstrating the binding characteristics of each specific Bla-Blip construct towards the appropriate target and a non-immunoglobulin negative control, *C. difficile* toxoid B. 10 µg mL^-1^ (125 nM) of the sensor construct were incubated with 20 µg mL^-1^ of target TmAb (135 nM) or toxoid B (74 nM). All data are presented as a mean of three repeats and error bars represent ±SEM.


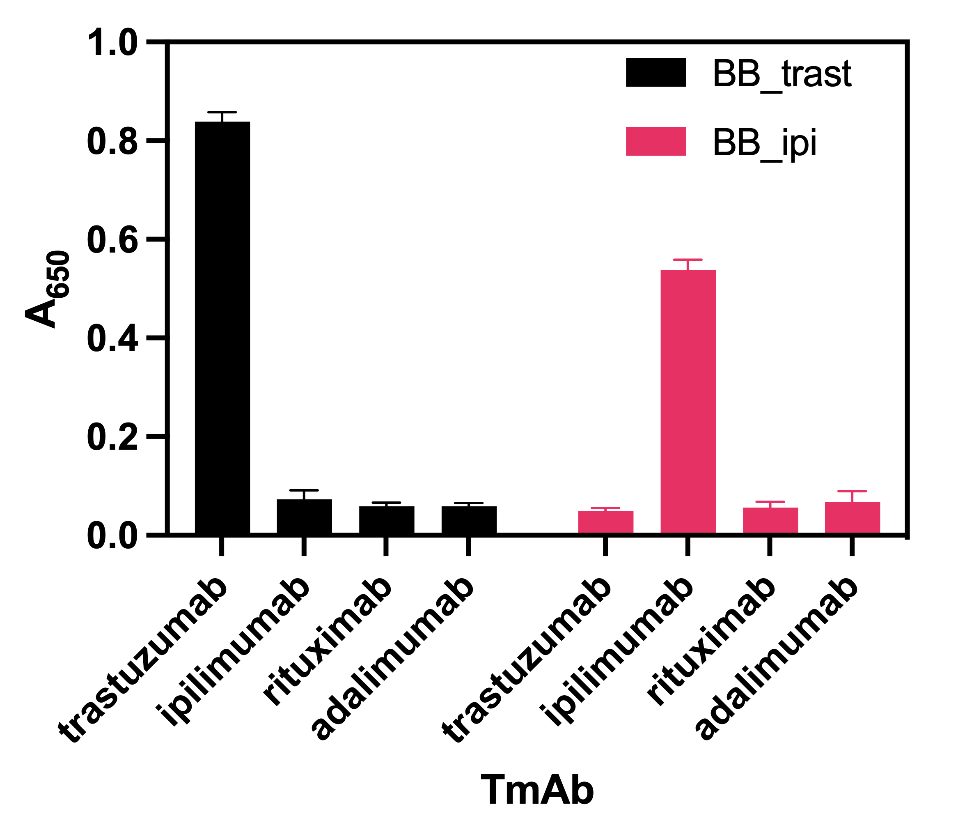
**Figure S5 TmAb BLA-BLIP sensors are specific to their target analyte**. Direct ELISAs were performed on BB_Trast and BB_Ipi to determine their specificity. 10 µg mL^-1^ (125 nM) of the sensor constructs were incubated with 20 µg mL^-1^ of specific or non-specific TmAb (135 nM). Data of biosensors binding to their respective targets and non-specific analytes are presented as a mean of three repeats and error bars represent ±SEM.


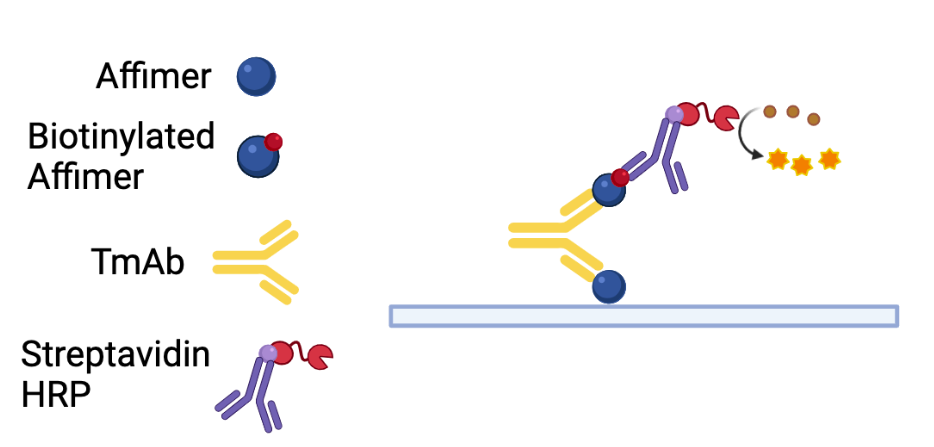


**Figure S6. Diagram of Bridge ELISA.** In the Bridge ELISA, unlabelled Affimer are first immobilised on the surface of the plate, incubated with the TmAb which are subsequently detected using a biotin- labelled Affimer of the same specificity. The symmetrical conformation of an IgG should allow for the binding of two anti-ID Affimers should they bind to the variable domain of the antibody.

**Table S1. K_D_ values calculated from evaluation of langmuir model fits of SPR curves.**

All anti-idiotypic Affimer® proteins have nM affinity for their respective TmAb analytes and are within ~ 12-fold of one another. K_D_ values were calculated with BIAevaltuation software and are presented as a mean of three replicates ±SEM. (Aff-Ipi – ipilimumab n=2).

|  | K_D_ (nM) | SEM (nM) |
| --- | --- | --- |
| Aff-Trast – trastuzumab | 0.75 | ± 0.12 |
| Aff-Ipi – ipilimumab (n=2) | 7.8 | ± 0.8 |
| Aff-Ada – adalimumab | 9.77 | ±2.54 |
| Aff-Rit – Rituximab | 4 | ±0.75 |

**Table S2. Interpolated standard curves for BB_Trast and BB_Ipi provided quantifiable ranges based on accuracy and precision of the sensors in spiked 1% human serum.**

Percentage recovery and percentage coefficient variance (%CV) values for BB_Trast and BB_Ipi were used to determine the quantifiable range of each sensor based on recovery values between 80 – 120% and % CV < 25%.

|  | BB_Ipi | | | BB_Trast | | | | |
| --- | --- | --- | --- | --- | --- | --- | --- | --- |
| mAb concentration | 30 pM | 100 pM | 300 pM | 30 pM | 100 pM | 300 pM | 1 nM | 3 nM |
| % recovery | 101 % | 101 % | 94 % | 81 % | 119 % | 83 % | 101 % | 105 % |
| % CV | 4.7 % | 2.7 % | 12 % | 12.3 % | 22 % | 13.2 % | 16.2 % | 17.6 % |

**DNA and Protein Sequences**

Within the BLA-BLIP sensor sequence the Affimer variable region sequences are denoted as XXX. The sequences and plasmids will be shared with any academic who does not have a commercial interest, under an MTA.

BB_TRAST/BB_IPI/BB_RIT/BB_ADA:

Red – leader sequence

Blue – His-tag

Green – thrombin cleavage site

Yellow highlight – TEM1-β-lactamase(E104D)

Light grey highlight – linker 1 (L1)

Pink – Anti-idiotype Affimer

Green highlight – semi-flexible linker 2 (L2)

Dark grey highlight – linker 3 (L3)

Cyan highlight – β-lactamase inhibitor protein(E31A)

Orange – Strep-tag

DNA:

ATGGCTTCTATCCAGCACTTCCGTGTTGCTCTGATCCCGTTCTTCGCTGCTTTCTGCCTGCCGGTTTTCGCTGCTCACCACCACCACCACCACCACCACCTGGTTCCGCGTGGTTCTCACCCGGAAACCCTGGTTAAAGTTAAAGACGCTGAAGACCAGCTGGGTGCTCGTGTTGGTTACATCGAACTGGACCTGAACTCTGGTAAAATCCTGGAATCTTTCCGTCCGGAGGAGAGGTTCCCGATGATGTCTACCTTCAAAGTTCTGCTGTGCGGTGCTGTTCTGTCTCGTGTTGACGCTGGTCAGGAACAGCTGGGTCGTCGTATCCACTACTCTCAGAACGACCTGGTTGACTACTCTCCGGTTACCGAAAAACACCTGACCGACGGTATGACCGTTCGTGAACTGTGCTCTGCTGCTATCACCATGTCTGACAACACCGCTGCTAACCTGCTGCTGACCACCATCGGTGGTCCGAAAGAACTCACTGCGTTCCTGCACAACATGGGTGACCACGTTACCCGTCTGGACCGTTGGGAACCGGAACTGAACGAAGCTATCCCGAACGACGAACGTGACACCACCATGCCGGCTGCTATGGCTACCACCCTGCGTAAACTGCTGACCGGTGAACTGCTGACCCTGGCTTCTCGTCAGCAGCTGATCGACTGGATGGAAGCTGACAAAGTTGCTGGTCCGCTGCTGCGTTCTGCTCTGCCGGCTGGTTGGTTCATCGCTGACAAATCTGGTGCTGGTGAACGTGGTTCTCGTGGTATCATCGCTGCTCTGGGTCCGGACGGTAAACCGTCTCGTATCGTTGTTATCTACACCACCGGTTCTCAGGCTACTATGGACGAACGTAACCGTCAGATCGCTGAAATCGGTGCTTCTCTGATCAAACACTGGACTAGTGCAAACTCCCTGGAAATCGAAGAACTGGCTCGTTTCGCTGTTGACGAACACAACAAAAAAGAAAACGCTCTGCTGGAATTCGTTCGTGTTGTTAAAGCGAAAGAACAGXXXXXXXXXXXXXXXXXXXXXXXXACCATGTACTACCTGACCCTGGAAGCTAAAGACGGTGGTAAAAAGAAACTGTACGAAGCGAAAGTTTGGGTTAAGXXXXXXXXXXXXXXXXXXXXXXXXXXXAACTTCAAAGAACTGCAGGAGTTCAAACCAGTAGTCGACGGTGGTTCTGGTGGTTCTGGTGGTTCTGGTGGTTCTGGTGGTTCTGGTGGTTCTGGTGCTGAAGCTGCTGCTAAAGAAGCTGCTGCTAAAGAAGCTGCTGCTAAAGAAGCTGCTGCTAAAGAAGCTGCTGCTAAAGAAGCTGCTGCTAAAGCTGGTTCTGGTGGTTCTGGTGGTTCTGGTGGTTCTGGTGGTTCTGGTGGTTCTGGTGCTGAAGCTGCTGCTAAAGAAGCTGCTGCTAAAGAAGCTGCTGCTAAAGAAGCTGCTGCTAAAGAAGCTGCTGCTAAAGAAGCTGCTGCTAAAGCTGGTTCTGGTGGTTCTGGTGGTTCTGGTGGTTCTGGTGGTTCTGGTGGTTCTGGTGGTGCTAGCAACTCCCTGGAAATCGAAGAACTGGCTCGTTTCGCTGTTGACGAACACAACAAAAAAGAAAACGCTCTGCTGGAATTCGTTCGTGTTGTTAAAGCGAAAGAACAGXXXXXXXXXXXXXXXXXXXXXXXXACCATGTACTACCTGACCCTGGAAGCTAAAGACGGTGGTAAAAAGAAACTGTACGAAGCGAAAGTTTGGGTTAAGXXXXXXXXXXXXXXXXXXXXXXXXXXXAACTTCAAAGAACTGCAGGAGTTCAAACCAGTAGCGGCCGCTGCTGGTGTTATGACCGGTGCTAAATTCACCCAGATCCAGTTCGGTATGACCCGTCAGCAGGTTCTGGACATCGCTGGTGCTGAAAACTGCGCTACTGGCGGTAGCTTCGGTGACTCTATACACTGCCGTGGTCACGCTGCTGGTGACTACTACGCTTACGCTACCTTCGGTTTCACCTCTGCTGCTGCTGACGCTAAAGTTGACTCTAAATCTCAGGAAAAACTGCTGGCTCCGTCTGCTCCGACCCTGACCCTGGCTAAATTCAACCAGGTTACCGTTGGTATGACCCGTGCTCAGGTTCTGGCTACCGTTGGTCAGGGTTCTTGCACCACCTGGTCTGAATACTACCCGGCTTACCCGTCTACCGCTGGTGTTACCCTGTCTCTGTCTTGCTTCGACGTTGACGGTTACTCTTCTACCGGTTTCTACCGTGGTTCTGCTCACCTGTGGTTCACCGACGGTGTTCTGCAGGGTAAACGTCAGTGGGACCTGGTTGGTGGTCTCGGTGGTTGGTCTCACCCGCAGTTCGAAAAA

Protein:

MASIQHFRVALIPFFAAFCLPVFAAHHHHHHHHLVPRGSHPETLVKVKDAEDQLGARVGYIELDLNSGKILESFRPEERFPMMSTFKVLLCGAVLSRVDAGQEQLGRRIHYSQNDLVDYSPVTEKHLTDGMTVRELCSAAITMSDNTAANLLLTTIGGPKELTAFLHNMGDHVTRLDRWEPELNEAIPNDERDTTMPAAMATTLRKLLTGELLTLASRQQLIDWMEADKVAGPLLRSALPAGWFIADKSGAGERGSRGIIAALGPDGKPSRIVVIYTTGSQATMDERNRQIAEIGASLIKHWTSANSLEIEELARFAVDEHNKKENALLEFVRVVKAKEQXXXXXXXXTMYYLTLEAKDGGKKKLYEAKVWVKXXXXXXXXXNFKELQEFKPVVDGGSGGSGGSGGSGGSGGSGAEAAAKEAAAKEAAAKEAAAKEAAAKEAAAKAGSGGSGGSGGSGGSGGSGAEAAAKEAAAKEAAAKEAAAKEAAAKEAAAKAGSGGSGGSGGSGGSGGSGGASNSLEIEELARFAVDEHNKKENALLEFVRVVKAKEQXXXXXXXXTMYYLTLEAKDGGKKKLYEAKVWVKXXXXXXXXXNFKELQEFKPVAAAAGVMTGAKFTQIQFGMTRQQVLDIAGAENCATGGSFGDSIHCRGHAAGDYYAYATFGFTSAAADAKVDSKSQEKLLAPSAPTLTLAKFNQVTVGMTRAQVLATVGQGSCTTWSEYYPAYPSTAGVTLSLSCFDVDGYSSTGFYRGSAHLWFTDGVLQGKRQWDLVGGLGGWSHPQFEK

**Tables of Primers**

**Table S3. Primers used to introduce Linker 1 (L1) to Affimer DNA**

| Primer | Sequence |
| --- | --- |
| Aff-Spe*I* | AACG*ACTAGT*AACTCCCTGGAAATCGAAGAACTG |
| Aff-Sal*I* | TAAT*GTCGAC*TACTGGTTTGAACTCCTGCAGTTCTTTG |
| Aff-Spe*I*-ASS | AACG*ACTAGT*GCAGCTTCAAGTAACTCCCTGGAAATCGAAGAACTG |

**Table S4. Primers used to introduce Linker 3 (L3) to Affimer DNA**

| Primer | Sequence |
| --- | --- |
| Aff-Nhe*I* | ATG*GCTAGC*AACTCCCTGGAAATCGAAG |
| Aff-Not*I* | TAAT*GCGGCCGC*TACTGGTTTGAACTCCTGCAGTTCTTTG |
| Aff-Not*I*-ASS | ATTA*GCGGCCGC*ACTTGAAGCTACTGGTTTGAACTCCTGCAGTTCTTTG |

**Table S5. Primers used to introduce longer Linker 2 (L2) to Affimer DNA**

| Primer | Sequence |
| --- | --- |
| Aff-Spe*I* | AACG*ACTAGT*AACTCCCTGGAAATCGAAGAACTG |
| Aff-Sal*I*-(GSG)_4_ | TAAT*GTCGAC*GCCAGACCCGCCAGAACCACCTGACCCA  CCGGAGCCTACTGGTTTGAACTCCTGCAGTTCTTTG |
| Aff-Nhe*I*-(GSG)_4_ | ATGA*GCTAGC*GGCTCCGGGGGTCAGGTGGTTCTGGCGG  GTCTGGCAACTCCCTGGAAATCGAAGAACTG |
| Aff-Not*I* | TAAT*GCGGCCGC*TACTGGTTTGAACTCCTGCAGTTCTTTG |

**Table S6. Primers used to introduce cysteine residues to Affimer DNA**

| Primer | Sequence |
| --- | --- |
| Aff-Nhe*I* | ATG*GCTAGC*AACTCCCTGGAAATCGAAG |
| Aff-Not*I* | TAAT*GCGGCCGC*TACTGGTTTGAACTCCTGCAGTTCTTTG |

**Table of Plasmids**

**Table S7. Plasmids containing four engineered anti-ID BLA-BLIP sensors**

| Plasmid Name | Template plasmid | Affimers |
| --- | --- | --- |
| pEEC01 | pET28a (+) | Aff-HER x2 |
| pEEC02 | pET28a (+) | Aff-IPI x2 |
| pEEC03 | pET28a (+) | Aff-RIT x2 |
| pEEC04 | pET28a (+) | Aff-ADA x2 |
| pET11a | pET11a | Affimer + C terminal Cys |
